# Superior thermotolerance in young versus adult rats undergoing heat stroke is associated with age-related differences in intestinal barrier integrity and heat shock protein responses

**DOI:** 10.3389/fcell.2026.1642359

**Published:** 2026-02-13

**Authors:** Yuanhao Cai, Lei Lei, Jikuai Chen, Xi Zhao, Juelin Chen, Jiawei Zhou, Yankun Pei, Yawei Wang, Yitong Gong, Jianyao You, Yangyang Cao, Muge Song, Jun Ma, Weiyi Ma, Meng Wang, Wenjun Chang, Qing Song, Lin Zhou, Lei Li, Shuogui Xu

**Affiliations:** 1 Department of Pediatrics, Changhai Hospital, Naval Medical University, Shanghai, China; 2 Department of Health Toxicology, Faculty of Naval Medicine, Naval Medical University, Shanghai, China; 3 Department of Pediatrics, Zhabei Central Hospital, Shanghai, China; 4 Department of Emergency and Trauma, Changhai Hospital, Naval Medical University, Shanghai, China; 5 Heatstroke Treatment and Research Center of PLA, Sanya, China; 6 Department of Critical Care Medicine, 923 Hospital of the PLA Joint Logistic Support Force, Nanning, China; 7 Department of Emergency, First Medical Center of Chinese PLA General Hospital, Beijing, China; 8 Department of Critical Care Medicine, Hainan Hospital, Chinese PLA General Hospital, Sanya, China; 9 Faculty of Naval Medicine, Naval Medical University, Shanghai, China; 10 Department of Emergency, The Second Naval Hospital of Southern Theater Command of PLA, Sanya, China

**Keywords:** core body temperature, heat shock proteins, heat stroke, intestinal barrier, thermotolerance

## Abstract

Heat stroke (HS) is a life-threatening condition exacerbated by rising global temperatures, with children identified as a particularly vulnerable population. Despite this, basic research on age-related differences in thermotolerance remains limited. In this study, we established a high-temperature and high-humidity exposure model with real-time core body temperature (CBT) monitoring to investigate thermotolerance in young versus adult rats. The results showed that young rats exhibited prolonged CBT plateau phases and delayed HS onset, indicating enhanced thermotolerance compared to adult rats. This was accompanied by significantly milder multi-organ injury and reduced intestinal barrier damage. Young rats displayed lower serum levels of D-lactate and intestinal fatty acid-binding protein, better-preserved intestinal epithelial ultrastructure, and higher expression of tight junction proteins such as ZO-1, Occludin, and E-cadherin. Moreover, young rats showed elevated expression of heat shock proteins (HSP40 and HSP70) in intestinal tissues, which likely contributed to improved barrier integrity and cellular protection. These findings suggest that enhanced intestinal barrier stability and robust HSP responses underlie the superior thermotolerance observed in young rats. However, despite their physiological advantages, infants and young children often suffer poor HS outcomes due to behavioral limitations and caregiver negligence, especially in enclosed environments such as parked vehicles. This highlights the critical need for enhanced caregiver awareness, improved pediatric emergency response training, and preventive strategies to mitigate pediatric HS risk.

## 1 Introduction

With the intensifying trend of global warming, human societies are increasingly threatened by heat stress (Khan and Mubeen, 2025; Bevacqua et al., 2025). When the body is subjected to sustained heat stress, thermoregulatory mechanisms may become decompensated, leading to excessive heat production relative to heat dissipation. As a result, core body temperature (CBT) rises rapidly, potentially culminating in the development of heat stroke (HS)—a life-threatening medical emergency (Li et al., 2023). HS is generally classified into two types based on etiology and affected populations: classical heat stroke (CHS) and exertional heat stroke (EHS). Children are considered a high-risk population for CHS (Xiang et al., 2024; Liu et al., 2020). In recent years, pediatric heat illness and HS have received increasing attention (Fisher et al., 2022). On the one hand, children are physiologically and anatomically distinct from adults, with immature organ systems and underdeveloped thermoregulatory mechanisms (Okada et al., 2025). On the other hand, children are particularly vulnerable to high-temperature environments due to common incidents such as being accidently left in enclosed vehicles (Sartin et al., 2025). As a result, many researchers have identified pediatric HS as an urgent and understudied clinical concern requiring further investigation (Marudo et al., 2025). Despite growing clinical interest, there is currently a lack of basic research exploring the differential thermotolerance between young and adult rats.

In our previous work, we established a stable high-temperature and high-humidity environment combined with a wireless CBT monitoring system in rats, which enabled real-time observation and analysis of thermoregulatory responses under heat stress (Li et al., 2024a). This work culminated in the formulation of the three-phase thermoregulatory response theory. Therefore, building upon our previously proposed three-phase thermoregulatory response theory, we employed a high-temperature, high-humidity exposure platform combined with real-time CBT monitoring to investigate age-related differences in thermotolerance between young and adult rats. Given our prior findings that intestinal barrier disruption acts as an initiating factor in the pathogenesis of HS and significantly contributes to its progression (Ma et al., 2025; Li et al., 2024b; Li et al., 2024c; Li et al., 2021), this study places particular emphasis on evaluating differences in intestinal barrier function between the two age groups, aiming to clarify its role in thermotolerance. Furthermore, our earlier work has shown that heat acclimation enhances intestinal expression of heat shock proteins (HSPs), thereby improving thermotolerance in rats (Li et al., 2024c). Building on this foundation, the present study integrates continuous CBT monitoring, histological assessment of intestinal barrier integrity, and analysis of HSPs expression in young and adult rats during HS induction. This comprehensive approach aims to elucidate the physiological and molecular mechanisms underlying the superior thermotolerance observed in young rats. This study provides novel insights into the age-related mechanisms of thermotolerance, with implications for the prevention and treatment of pediatric HS.

## 2 Materials and methods

### 2.1 Animals and treatment

Male adult Sprague-Dawley (SD) rats (7–9 weeks old) and young SD rats (3–5 weeks old) were obtained from Sippr B&K Laboratory Animal Ltd. (Shanghai, China). The animals were housed in the Specific Pathogen-Free (SPF) Animal Experiment Center of the Naval Medical University and acclimated for 1 week under controlled conditions: temperature 22 °C ± 1 °C, relative humidity 50% ± 5%, and a 12-h light/dark cycle. All experimental procedures were approved by the Institutional Animal Ethics Committee of the Naval Medical University (Approval No. NMUMREC-2021–002) and conducted in accordance with the NIH Guide for the Care and Use of Laboratory Animals. Standard pellet rat chow and distilled tap water were provided *ad libitum*. Rats were classified as young (3–5 weeks) and adult (7–9 weeks), consistent with previous study employing similar age ranges (Wang et al., 2013).

### 2.2 Heat stroke protocol and sample collection

**FIGURE 1:**
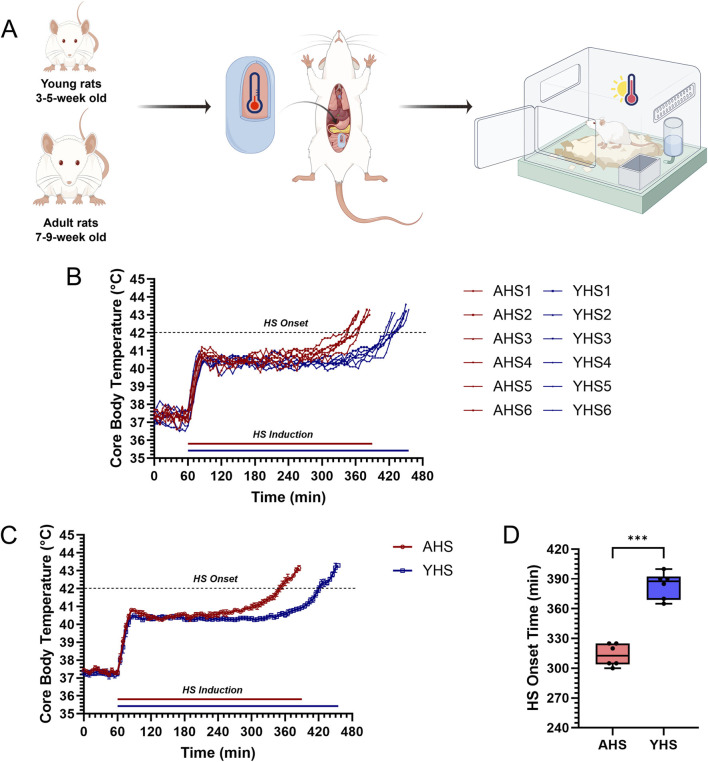
Experimental design and thermoregulatory response characteristics in HS rats. **(A)** Young (3–5 weeks) and adult (7–9 weeks) rats were implanted with CBT (Tc) monitoring capsules and exposed to a high-temperature, high-humidity chamber to induce HS. **(B)** Tc curves of individual HS rats in each group during HS induction. **(C)** Mean Tc curves of the two groups at each time point during HS induction. **(D)** Comparison of the time to HS onset between the two groups. The data in **(B)** are presented as line graphs. Data in **(C)** are shown as mean ± SEM (n = 6 per group). Data in **(D)** are displayed as box-and-whisker plots (n = 6 per group). ***P < 0.001.

The HS induction protocol was performed in accordance with our previously established methods. A total of 24 rats were randomly assigned to four groups based on age and body weight: adult control (AC), young control (YC), adult heat stroke (AHS), and young heat stroke (YHS), with six rats in each group (Figure 1A). As described in earlier studies (Li et al., 2024a; Li et al., 2024c; Li et al., 2021), prior to HS induction, temperature-monitoring capsules (SV223, Flamingo Technology Co., Ltd., Shanghai, China) were implanted in rats from the AHS and YHS groups to continuously measure CBT (Tc) at 5-min intervals. Rats in the control groups (AC and YC) underwent sham surgeries. Briefly, each rat was anesthetized with isoflurane, and a sterilized temperature-monitoring capsule (1.5 cm × 0.5 cm, 2 g) was surgically implanted into the abdominal cavity through a small aseptic incision. Rats were allowed to recover for 24 h prior to heat exposure. HS induction was then carried out by placing the rats in an artificial climate chamber (LTH-575N-01, Drawell Scientific Instrument Co., Ltd., Shanghai, China) maintained at 40 °C ± 1 °C and 60% ± 5% relative humidity. Core body temperature and physical condition were monitored in real time. Once a rat’s CBT exceeded 42 °C accompanied by signs of unconsciousness, it was immediately removed from the chamber and placed in a room-temperature environment. Three hours later, samples including blood and organ tissues were collected for further analysis.

### 2.3 Blood sample examination

Serum biochemical indices, including blood urea (BU), creatine kinase (CK), creatinine (CREA), alanine aminotransferase (ALT), and aspartate aminotransferase (AST), were measured using an automatic biochemical analyzer (HITACHI 7080, Tokyo, Japan). Plasma levels of D-Lactate and intestinal fatty acid-binding protein (I-FABP) were quantified using a D-Lactate colorimetric assay kit (ab83429, Abcam, Cambridge, United Kingdom) and an intestinal fatty acid-binding protein (IFABP) ELISA kit (KE00263, Proteintech, Shanghai, China), respectively, following the manufacturers’ instructions. Absorbance was detected using a SpectraMax M2e microplate spectrophotometer (Bio-Rad, Berkeley, CA, United States), and concentrations were calculated based on standard curves generated for each assay.

### 2.4 Western blot analysis

Intestinal tissues from adult and young rats were rinsed with PBS (Bio-Light, P1022, Shanghai, China) and cut into 1 cm segments. The samples were snap-frozen in liquid nitrogen and stored at −80 °C until further analysis. For protein extraction, tissues were homogenized in lysis buffer (Beyotime Institute of Biotechnology, P0013B, Shanghai, China) supplemented with protease and phosphatase inhibitor cocktails (Roche Diagnostics GmbH, 04906837001/5892970001, Germany) and grinding beads (Servicebio, G0203, Wuhan, China). Homogenization was performed for 10 min, followed by centrifugation at 15,294 × g for 10 min at 4 °C. The supernatant was collected, and protein concentration was quantified using a BCA assay kit (Epizyme Biotech, ZJ103, Shanghai, China). Samples were diluted to a final concentration of 2 μg/μL. Equal amounts of protein (20 μg) were separated by 4%–12% SurePAGE™ gels (GenScript, M00654, Nanjing, China) and transferred to polyvinylidene fluoride (PVDF) membranes (Millipore, ISEQ00010, Germany) using an eBlot™ L1 Fast Wet Transfer System (GenScript, L00686C, Nanjing, China). Membranes were blocked with quick blocking buffer (Beyotime Institute of Biotechnology, P0252, Shanghai, China) for 1 h at room temperature, followed by overnight incubation at 4 °C with the following primary antibodies: ZO-1 (Proteintech, 21773-1-AP, rabbit, 1:5,000), E-cadherin (Proteintech, 20874-1-AP, rabbit, 1:20,000), Occludin (Proteintech, 27260-1-AP, rabbit, 1:5,000), HSP90 (Proteintech, 13171-1-AP, rabbit, 1:6,000), HSP70 (Proteintech, 10995-1-AP, rabbit, 1:10,000), HSP60 (Proteintech, 15282-1-AP, rabbit, 1:6,000), HSP40 (Proteintech, 13174-1-AP, rabbit, 1:10,000), and GAPDH (Proteintech, 60004-1-Ig, mouse, 1:50,000). After washing, membranes were incubated with HRP-conjugated secondary antibodies against rabbit or mouse IgG (Abways, AB0101/AB0102, Shanghai, China) at a dilution of 1:8,000 for 2 h at room temperature. Protein bands were visualized using the Omni-ECL™ ultra-sensitive chemiluminescence kit (Epizyme Biotech, SQ201, Shanghai, China) and imaged using an Amersham Imager 600 (GE Healthcare Bio-Sciences AB). Quantification of Western blot bands from three biological replicates was performed using ImageJ software (NIH, version 1.52e).

### 2.5 Histological examination

All organ tissue samples were processed following the protocol described in our previous study (Li et al., 2024d). Briefly, tissues were fixed, dehydrated, embedded in paraffin, sectioned, and stained with hematoxylin and eosin (HE). The stained sections were examined under a Leica DM2000 light microscope (Wetzlar, Germany) and digitized using a Panoramic MIDI slide scanner (3DHISTECH, Hungary) for detailed histological analysis. For transmission electron microscopy (TEM), intestinal samples were fixed in 1% osmium tetroxide (OsO_4_) in 0.1 M phosphate-buffered saline (PBS, pH 7.4) for 2 h at room temperature, followed by dehydration, embedding, polymerization, and sectioning into 60–80 nm ultrathin slices. The sections were then stained with 2% uranyl acetate and 2.6% lead citrate and examined using a Hitachi-7000 electron microscope (Naka, Japan). For immunofluorescence staining, paraffin-embedded intestinal sections from adult and young rats were dewaxed and rinsed repeatedly with distilled water, followed by antigen retrieval under high pressure for 30 min. After washing with PBS (Bio-Light, P1022, Shanghai, China), sections were blocked at room temperature in the dark for 30 min. Primary antibodies against E-cadherin, Occludin, and ZO-1 were sequentially applied, followed by corresponding secondary antibodies and TSA fluorophores. Antigen retrieval and microwave-mediated antibody stripping (15 min) were performed between each round of staining to enable multiplex labeling. The primary antibodies used were E-cadherin (mouse, 1:2000; Servicebio, GB12083), Occludin (rabbit, 1:2000; Servicebio, GB111401), and ZO-1 (rabbit, 1:2000; Servicebio, GB115686). The TSA dyes used were iF488-Tyramide, iF647-Tyramide, and iF546-Tyramide (Servicebio, G1231/G1232/G1251, Wuhan, China), and the secondary antibodies were HRP-conjugated goat anti-rabbit IgG and goat anti-mouse IgG (Servicebio, GB23303/GB23301, Wuhan, China). Nuclei were counterstained with DAPI (Servicebio, G1012, Wuhan, China) for 15 min and mounted with an anti-fade reagent (Servicebio, G1401, Wuhan, China). Fluorescent images were captured using a Nikon Eclipse TI-SR fluorescence microscope (Nikon, Japan).

### 2.6 Statistical analysis

Data are presented as mean ± standard error of the mean (SEM). Statistical analyses were performed using GraphPad Prism (version 10.3.1; GraphPad Software, CA, United States). Comparisons among multiple groups were conducted using one-way analysis of variance (ANOVA) followed by the Tukey-Kramer post hoc test. For comparisons between two independent groups, a two-tailed Student’s t-test was employed. A P-value ≤0.05 was considered indicative of statistical significance.

## 3 Results

### 3.1 Young rats exhibit enhanced thermotolerance during HS induction compared to adult rats

CBT of adult and young rats was continuously monitored using temperature-sensing capsules implanted in the abdominal cavity. Based on our previously established three-phase thermoregulatory response model under heat stress (Li et al., 2024a), there was no significant difference in the rate of temperature increase during the initial rising phase between adult and young rats (Figures 1B,C). Similarly, the peak CBT achieved during the plateau phase did not differ significantly between the two groups (Figures 1B,C). However, the duration of the plateau phase was significantly longer in young rats than in adult rats. Further analysis revealed that the onset time of HS was significantly delayed in young rats (Figure 1D). These results indicate that young rats exhibit greater thermotolerance, as evidenced by their ability to maintain plateau-phase CBT for a longer period and delay the onset of HS.

### 3.2 Young rats exhibit milder multi-organ damage after HS compared to adult rats

**FIGURE 2:**
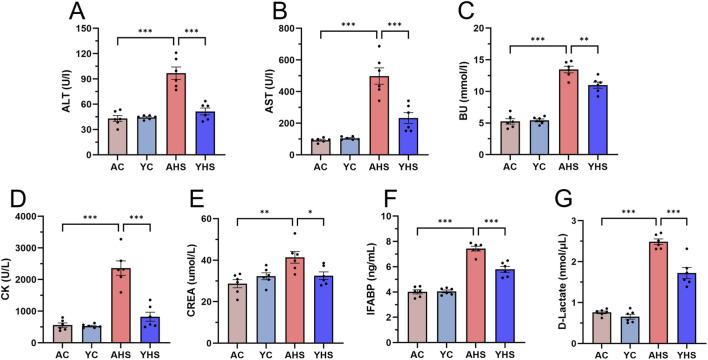
Serum biochemical and intestinal injury markers reveal that young rats experienced less severe organ damage following HS compared to adult rats. Blood samples were collected from rats in each group 3 h after HS induction. Serum levels of biochemical markers including ALT **(A)**, AST **(B)**, BU **(C)**, CK **(D)**, and CREA **(E)** were measured using an automated biochemical analyzer. Serum levels of intestinal injury markers, including IFABP **(F)** and D-lactate **(G)**, were determined using ELISA kits. Data are presented as mean ± SEM (n = 6 per group). *P < 0.05, **P < 0.01, ***P < 0.001.

**FIGURE 3:**
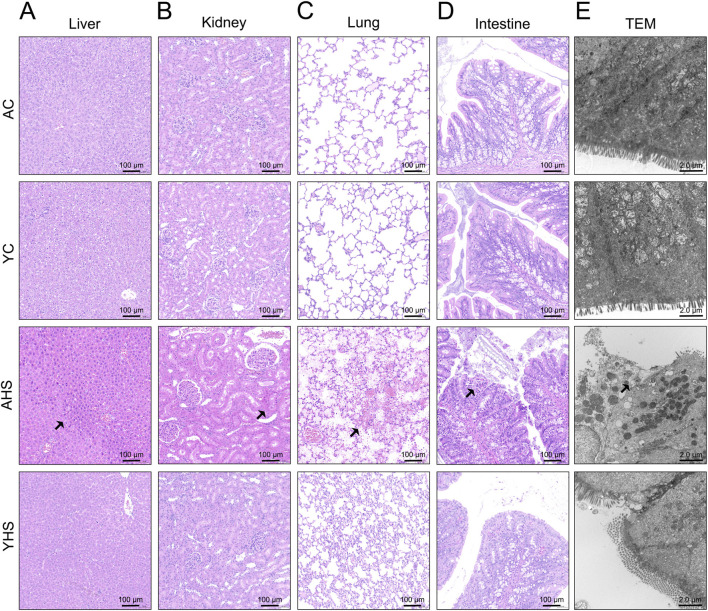
Histological and ultrastructural analyses reveal attenuated multi-organ injury in young rats compared to adults after HS. **(A–D)** Representative HE images (scale bar, 100 μm) of the liver **(A)**, kidney **(B)**, lung **(C)**, and intestine **(D)**. Arrows indicate representative regions of tissue structural disruption and inflammatory involvement. **(E)** Representative TEM images of the intestinal barrier (scale bar, 2.0 μm). n = 3 per group.

One of the key clinical manifestations of HS is heat stress-induced multi-organ damage. Our previous studies have demonstrated that within 3 h after HS onset, significant evidence of multi-organ injury can be detected through serum biochemical markers and histopathological analysis (Li et al., 2024c; Li et al., 2021). In the present study, adult rats exhibited marked elevations in biochemical indicators (Figures 2A–E) and characteristic pathological changes in major organs (liver, kidney, lung, and intestine) at 3 h post-HS induction (Figures 3A–D). Specifically, liver sections from AHS rats showed extensive parenchymal injury characterized by disruption of hepatic architecture, hepatocellular degeneration and necrosis, vascular dilation and congestion within portal areas, and inflammatory cell infiltration, rather than discrete nuclear morphological alterations. Mild granulocytic infiltration was observed around the bile ducts, together with dilation and congestion of portal vessels, accompanied by inflammatory cell accumulation. The central veins and adjacent hepatic sinusoids also appeared dilated and congested, consistent with acute hepatic injury. In the kidneys, AHS rats exhibited prominent glomerular and interstitial vascular congestion with inflammatory cell infiltration. Renal tubular epithelial cells showed marked swelling, luminal narrowing, and structural degeneration, consistent with acute tubular injury. Lung tissues from AHS rats revealed widespread alveolar wall edema with moderate thickening, narrowed alveolar spaces, and infiltration of mononuclear and granulocytic cells. Pulmonary capillaries were dilated and congested, and regions of atelectasis and emphysema were observed, together with focal pulmonary hemorrhage, reflecting acute lung injury. Intestinal sections showed severe mucosal injury in AHS rats, characterized by extensive epithelial disruption and sloughing, marked vascular congestion, and pronounced villus structural damage, including villus shortening, fragmentation, or detachment, rather than specific nuclear changes. These representative features are indicated by arrows in the AHS liver sections. In contrast, YHS rats exhibited markedly attenuated pathological changes across all examined organs. Liver tissues from YHS rats displayed largely preserved lobular architecture, with only scattered hepatocyte degeneration and minimal necrosis. In the kidneys, glomerular and tubular structures remained largely intact, with only mild vascular congestion and occasional inflammatory infiltration. Pulmonary tissues displayed mild alveolar wall edema and limited inflammatory cell infiltration, without evidence of hemorrhage or emphysema. Intestinal histology revealed partial villus blunting and mild epithelial shedding, while overall mucosal architecture was preserved, with significantly less congestion and structural disruption compared to AHS rats. These findings collectively suggest that young rats experience significantly milder multi-organ damage following HS, further supporting their enhanced thermotolerance under heat stress.

### 3.3 Young rats exhibit less intestinal barrier disruption after HS compared to adult rats

**FIGURE 4:**
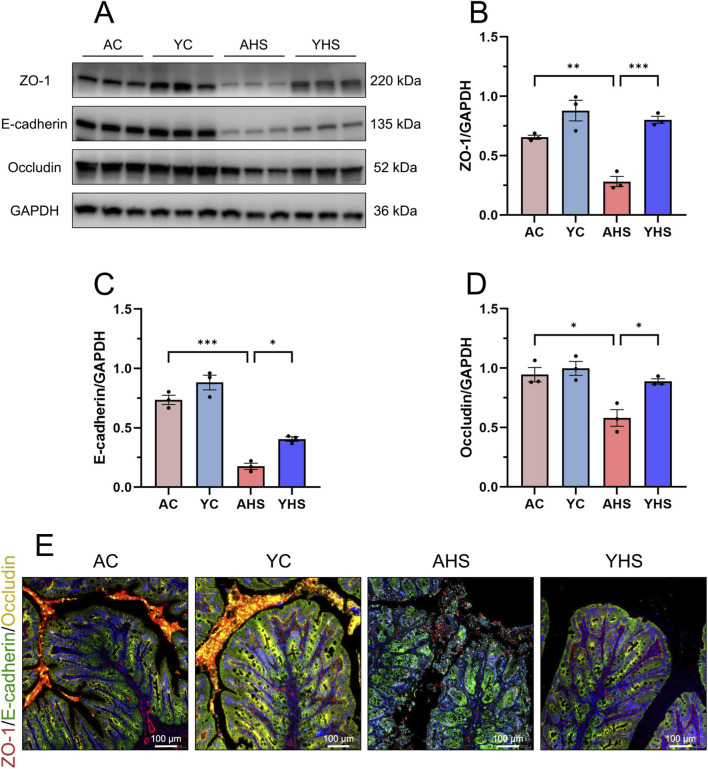
Western blot and immunofluorescence analyses reveal that young rats exhibit less disruption in the expression and distribution of intestinal barrier proteins compared to adult rats following HS. **(A)** Representative western blots showing ZO-1, E-cadherin, and Occludin expression after HS. GAPDH was used as the loading control. **(B–D)** Quantification of band intensities for ZO-1 **(B)**, E-cadherin **(C)**, and Occludin **(D)**, normalized to GAPDH. **(E)** Cell nuclei were stained with DAPI (blue), and ZO-1 (red), E-cadherin (green), and Occludin (yellow) were visualized using specific antibodies (scale bar, 100 μm). Data are presented as mean ± SEM, n = 3 per group. *P < 0.05, **P < 0.01, ***P < 0.001.

Our previous studies, along with recent advances in the field, have established that intestinal barrier disruption is an initiating event and a key pathophysiological driver in the development of HS (Dmytriv and Storey, 2024). Protecting the integrity of the intestinal barrier has been shown to effectively mitigate the onset and progression of HS-related organ damage. Therefore, we further evaluated intestinal barrier function in young rats following HS induction. Serum levels of D-lactate and IFABP, two established biomarkers of intestinal injury, were significantly elevated in adult rats at 3 h post-HS. However, the increase in YHS rats was markedly lower (Figures 2F,G). As described above, HE staining revealed prominent intestinal mucosal damage in AHS rats, while the extent of injury in YHS rats was substantially milder (Figure 3D). TEM further confirmed these findings (Figure 3E). In AHS rats, intestinal epithelial cells exhibited severe ultrastructural damage, including sparse and disorganized microvilli, partial microvillus detachment, and reduced villus height. The epithelial cells showed marked edema, with low electron density areas in the cytoplasm. Mitochondria were abundant but moderately swollen, with faded matrices, partial cristae loss, and ruptured outer membranes. Rough endoplasmic reticulum was dilated, with ribosome detachment. Numerous transport vesicles and scattered glycogen granules were observed, along with autophagolysosomes. Tight junctions between cells were disrupted or absent, with reduced tension filaments in the junctional complexes, loss of visible desmosomes, and disappearance of associated microfilaments and microtubules. In contrast, the ultrastructural damage in YHS rats was significantly less pronounced, with better preservation of microvillus structure, epithelial integrity, and intercellular junctions. To further evaluate barrier integrity, we assessed the expression of key tight junction proteins, including ZO-1, E-cadherin, and Occludin. Immunofluorescence and Western blot analyses revealed that young rats showed better preservation of tight junction proteins after heat stress. Following HS induction, expression levels of ZO-1, E-cadherin, and Occludin remained significantly higher in YHS rats than in AHS rats (Figures 4A–D). Immunofluorescence staining confirmed superior continuity and localization of these proteins in YHS rats, whereas AHS rats showed extensive discontinuity and disorganization (Figure 4E; Supplementary Figure S1). Together, these findings indicate that young rats possess better intestinal barrier integrity, which is more effectively maintained following HS. This enhanced barrier protection likely limits bacterial translocation and endotoxin leakage into the circulation, thereby reducing the severity of HS-associated multi-organ injury.

### 3.4 Elevated intestinal HSPs expression in young rats compared to adults contributes to enhanced thermotolerance after HS

**FIGURE 5:**
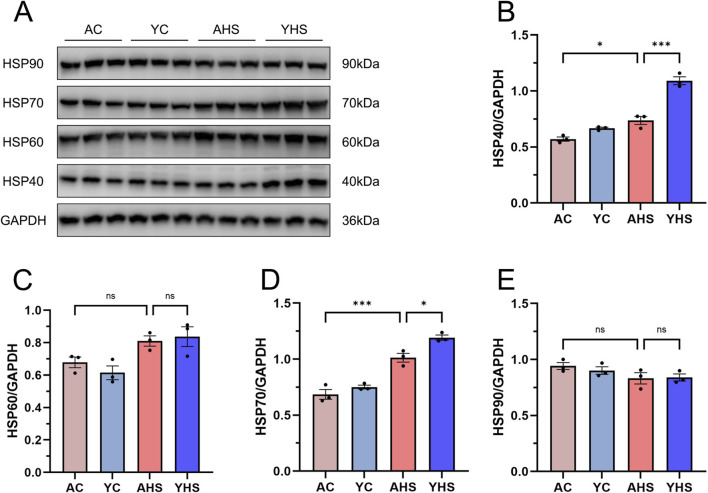
Young rats exhibit significantly higher expression of HSPs in intestinal tissues compared to adult rats after HS. **(A)** Representative Western blots of HSP40, HSP60, HSP70, and HSP90. GAPDH was used as the loading control. **(B–E)** Quantification of HSP40 **(B)**, HSP60 **(C)**, HSP70 **(D)**, and HSP90 **(E)** expression levels normalized to GAPDH. Data are presented as mean ± SEM, n = 3 per group. *P < 0.05, **P < 0.01, ***P < 0.001.

Previous studies from our team have demonstrated that heat acclimation can enhance thermotolerance by upregulating the expression of intestinal HSPs (Li et al., 2024c). Building on these findings, we further assessed the expression levels of representative HSPs—HSP90, HSP70, HSP60, and HSP40—in both adult and young rats following HS induction (Figure 5A). In the AHS group, HS induction led to a significant upregulation of HSP40 and HSP70, with statistically significant increases compared to control levels (Figures 5B,D). A mild increase in HSP60 expression was observed, though it did not reach statistical significance (Figure 5C). No notable changes were detected in HSP90 expression post-HS induction (Figure 5E). These results suggest a selective activation of specific HSP family members in response to acute heat stress. Importantly, in the YHS group, the expression levels of HSP40 and HSP70 were significantly higher than those observed in the AHS group under the same HS conditions. This indicates that young rats possess a more robust heat shock protein response when exposed to severe heat stress. Taken together, these findings suggest that the superior intestinal barrier integrity observed in YHS rats may be attributable, at least in part, to their enhanced capacity to induce protective HSPs such as HSP40 and HSP70. The stronger induction of these proteins in young rats likely contributes to improved cellular protection, protein homeostasis, and stabilization of epithelial structures, thereby mitigating HS-induced intestinal damage and subsequent systemic injury.

## 4 Discussion

With the intensifying trend of global warming, HS induced by extreme heat stress has drawn increasing attention from the medical research community. Our team, as the Heatstroke Treatment and Research Center of PLA, led the development and publication of China’s first official guidelines for the prevention and management of HS. CHS commonly occurs in vulnerable populations such as young children, pregnant women, the elderly, and individuals with chronic illnesses or compromised immune function. Although numerous studies support the adverse effects of heat stress on child health, critical knowledge gaps remain, including the identification of specific temperature thresholds, vulnerable populations, and the mechanisms underlying long-term impacts (Principi et al., 2025). Despite the clinical occurrence of HS in young children being well documented (Fisher et al., 2022), basic research on its underlying mechanisms in this population remains scarce. These gaps underscore the urgent need for mechanistic studies focused on pediatric susceptibility to HS. Studies have suggested that children differ significantly from adults in several physiological aspects, including thermoregulatory capacity, sweat gland development, and the ratio of body surface area to body mass (Marudo et al., 2025). These differences render them more susceptible to heat-related illnesses under high-temperature conditions, such as HS, dehydration, electrolyte imbalances, and cardiovascular stress responses. Moreover, infants and preschool-aged children are particularly vulnerable due to their limited ability to self-regulate behavior and their reliance on caregivers, which further increases their risk of exposure to harmful environments. Researchers and existing guidelines consistently emphasize that children differ significantly from adults in both physiological and morphological characteristics, resulting in distinct thermoregulatory responses. Specifically, children have a larger surface area-to-body mass ratio, higher metabolic rate, lower cardiac output, richer cutaneous blood flow, and lower sweating capacity. These traits collectively contribute to a traditionally held view that children possess less efficient thermoregulation, making them more susceptible to heat-related illnesses such as HS (Fisher et al., 2022; Okada et al., 2025; Marudo et al., 2025), (Krishna et al., 2024; Smith, 2019; Dunn and Kim, 2017). However, upon further literature review, we found no existing studies that have definitively demonstrated differences in thermotolerance between young and adult rats.

We initially hypothesized that, due to their ongoing physiological development and incomplete thermoregulatory mechanisms, young rats might exhibit a higher susceptibility to hyperthermia and HS than adult rats. Building on our previous work that defined the three-phase thermoregulatory response in rats exposed to heat stress (Li et al., 2024a), this study aimed to explore how this pattern differs between young and adult rats. Surprisingly, analysis of CBT under equivalent heat stress conditions revealed that young rats demonstrated superior thermotolerance relative to adult rats (Figures 1B–D). Young rats exhibit greater thermotolerance than adult rats, as evidenced by a longer plateau-phase CBT duration and delayed HS onset despite similar initial temperature rise rates and peak CBT levels. After repeated experimental validation confirmed this finding, we proceeded to further investigate the differences in thermotolerance between young and adult rats. Comparative analysis of blood samples and organ tissues from AHS and YHS rats revealed that young rats sustained significantly milder multi-organ damage after HS induction compared to adult rats (Figures 2, 3). This evidence suggests that young rats not only have a superior ability to maintain CBT compared to adult rats, but also exhibit greater organ thermotolerance to hyperthermia under equivalent heat stress conditions. Based on this evidence, we refuted our initial hypothesis and confirmed that young rats possess greater thermotolerance compared to adult rats, prompting further investigation into the underlying mechanisms. As mentioned in the introduction, our previous studies have demonstrated that intestinal barrier dysfunction plays a critical role in the pathogenesis of HS. Heat stress-induced impairment of the intestinal barrier may increase intestinal permeability and thereby potentially facilitate the translocation of gut bacteria, including endotoxins, into the circulation, which has been implicated in the systemic inflammatory response and multi-organ injury observed in HS (Tang et al., 2023; Epstein and Yanovich, 2019). Interventions using dietary supplements such as traditional Chinese herbs and probiotics to enhance the heat tolerance of the intestinal barrier have been shown to significantly alleviate barrier damage during HS and mitigate disease severity. Therefore, we further confirmed that young rats exhibited less intestinal barrier damage and better structural integrity compared to adult rats following the same HS induction. This conclusion was supported by multiple assessments, including the measurement of intestinal injury biomarkers in blood samples (Figures 2F,G), ultrastructural analysis of intestinal tissue via TEM (Figure 3E), and evaluation of tight junction protein expression and localization using Western blot and immunofluorescence (Figure 4). In our previous rat study on heat acclimation, we found that HSPs, including HSP90, HSP70, HSP60, and HSP40, played a pivotal role in enhancing intestinal thermotolerance and mitigating HS-induced damage (Li et al., 2024c; Gupta et al., 2017). Heat acclimation combined with probiotics-based ORS supplementation significantly upregulated the expression of these HSPs, which contributed to the preservation of tight junction integrity, reduction of intestinal injury biomarkers, and maintenance of ultrastructural stability of the intestinal barrier under heat stress. These findings highlight HSPs as key mediators of the protective effects of heat acclimation, supporting improved barrier function and systemic tolerance during HS. Therefore, we further examined the expression of HSPs in the intestinal tissue of young rats and found that the levels of HSP70 and HSP40 were significantly higher in young rats than in adult rats during HS induction (Figure 5). This finding partly explains the enhanced intestinal thermotolerance observed in young rats. Based on the above experimental findings, we confirmed that young rats exhibit enhanced thermotolerance and reduced severity of HS compared to adult rats, primarily attributed to better preservation of intestinal barrier integrity and elevated expression of HSPs. Future work in our laboratory will focus on elucidating the coordinated regulation of HSP70 and its co-chaperones, including HSPH1, to establish a more complete mechanistic framework underlying age-dependent differences in thermotolerance. In addition, upcoming studies will incorporate targeted inhibition or genetic manipulation of specific heat shock proteins to rigorously determine their causal roles in maintaining intestinal barrier integrity and enhancing tolerance to heat stress.

Although basic research has demonstrated that young rats exhibit superior thermotolerance compared to adult rats, clinical case reports and established guidelines consistently categorize children as a high-risk group for HS. This apparent contradiction between experimental findings and clinical perspectives prompted us to conduct a comprehensive review of the literature. We observed that, in contrast to adult-onset HS, pediatric HS is more commonly attributable to non-physiological factors (Sartin et al., 2025). Specifically, infants and young children have limited autonomous behavioral capacity and are highly dependent on caregivers. When caregivers lack adequate awareness of HS risks, children may be passively exposed to prolonged heat stress in high-temperature environments, ultimately leading to the onset of HS. Pediatric HS can be classified according to its underlying causes into three main types: enclosed heat syndrome, where excessive wrapping and covering of the child—including the head and face—leads to impaired heat dissipation and severe hypoxia; covered heat syndrome, involving over-bundling without hypoxia, resulting in heat accumulation; and HS due to confinement, which occurs when a child is left in a sealed or poorly ventilated space, such as a motor vehicle (Hammett et al., 2021). These patterns reflect the fact that pediatric HS often stems from passive heat exposure related to caregiver behavior rather than intrinsic thermoregulatory dysfunction. A study reported that since 1998, approximately 1,000 children in the United States have died from pediatric vehicular HS (PVH) after being unintentionally left in parked vehicles (Sartin et al., 2025). Despite numerous public awareness campaigns, the incidence of PVH has not markedly declined. Caregivers generally lack accurate perception of PVH risks, and existing prevention efforts have shown limited effectiveness. PVH fatalities primarily result from three scenarios: in approximately 52% of cases, a caregiver unintentionally forgets the child in the vehicle; around 25% involve children gaining access to an unattended vehicle on their own; and in about 21% of cases, the child is deliberately left in the vehicle by a caregiver, typically without intent to cause harm. The remaining 2% of cases have unknown circumstances (Null, 2025). Another study analyzed 541 pediatric deaths from vehicular heatstroke in the United States between 1990 and 2016, revealing that the majority of cases (78.2%) occurred when caregivers unintentionally left children in the vehicle, while only 16.6% involved children who were intentionally left behind without the caregiver anticipating the risk (Hammett et al., 2021). A forensic review revealed that the primary fatal scenarios of PVH fall into three categories: the most common being a caregiver unintentionally forgetting the child in the vehicle; the child independently entering and becoming trapped in the vehicle; and a caregiver intentionally leaving the child in the car for a short period without anticipating the heat-related risks. Most of these cases are classified as involuntary manslaughter due to negligence (Bushong and Diao, 2022). Therefore, through literature review, we found that pediatric HS is often caused by preventable environmental factors, particularly inadequate caregiving, being left in vehicles, or exposure to high-temperature settings. However, due to limited caregiver awareness, once pediatric HS occurs, it often progresses rapidly in infants and young children, presenting with severe clinical manifestations and resulting in high rates of mortality and long-term disability (Principi et al., 2025). In addition, a nationwide survey study conducted in Israel evaluated the knowledge, clinical practices, and resource preparedness of pediatric emergency department healthcare providers regarding the recognition and management of pediatric HS (Cohen-Ronen et al., 2021). The study revealed that most pediatric medical staff lacked adequate knowledge of optimal cooling methods, highlighting a significant gap between theoretical understanding and practical response to heat stroke in Israeli pediatric emergency settings.

In summary, the current clinical reality of pediatric HS—marked by high risk and poor outcomes in infants and young children—is likely attributable to two key factors: a lack of caregiver awareness regarding risk factors, and a significant gap between pediatric healthcare providers’ knowledge and their practical preparedness for managing pediatric HS cases. Particular attention should be given to PVH incidents caused by caregivers unintentionally leaving children in vehicles. In addition to basic public awareness education, a more structured risk intervention system should be developed. This includes the integration of legal measures, technological solutions, and social support. Preventive efforts should focus on advancing in-vehicle child presence detection systems and mandating the installation of high-temperature alert devices in all new vehicles. In addition, further refinement of pediatric HS treatment guidelines and the implementation of regular training and education programs for pediatric physicians on appropriate management strategies are also critically important.

## 5 Conclusion

In conclusion, the present study demonstrated that young rats exhibit greater thermotolerance compared to adult rats. This enhanced thermotolerance in young rats may be attributed to a more robust expression of HSPs in the intestinal tissue under heat stress, which likely contributes to better preservation of intestinal barrier integrity. It is important to note that although rat experiments suggest that young rats possess greater thermotolerance, infants and young children remain highly vulnerable to pediatric HS due to developmental immaturity and limited self-protective abilities. As a result, early recognition and timely intervention are often delayed, and most cases are already severe by the time they are discovered, leading to poor outcomes. Therefore, greater efforts must be directed toward educating caregivers and providing targeted training for pediatric healthcare providers.

## Data Availability

The raw data supporting the conclusions of this article will be made available by the authors, without undue reservation.

## Supplementary material

SUPPLEMENTARY FIGURE S1 Single-channel immunofluorescence images of intestinal barrier proteins after HS. Representative single-channel immunofluorescence images derived from the same multiplex-stained intestinal sections shown in Figure 4E. Cell nuclei were stained with DAPI (blue), while ZO-1 (red), E-cadherin (green), and Occludin (yellow) were detected using specific antibodies in separate channels (scale bar, 100 μm).
