# Supplementary figures and images for: Superior thermotolerance in young versus adult rats undergoing heat stroke is associated with age-related differences in intestinal barrier integrity and heat shock protein responses
Source: Front Cell Dev Biol. 2026 Feb 13;14:1642359. doi: 10.3389/fcell.2026.1642359 (PMC12946125; doi:10.3389/fcell.2026.1642359)

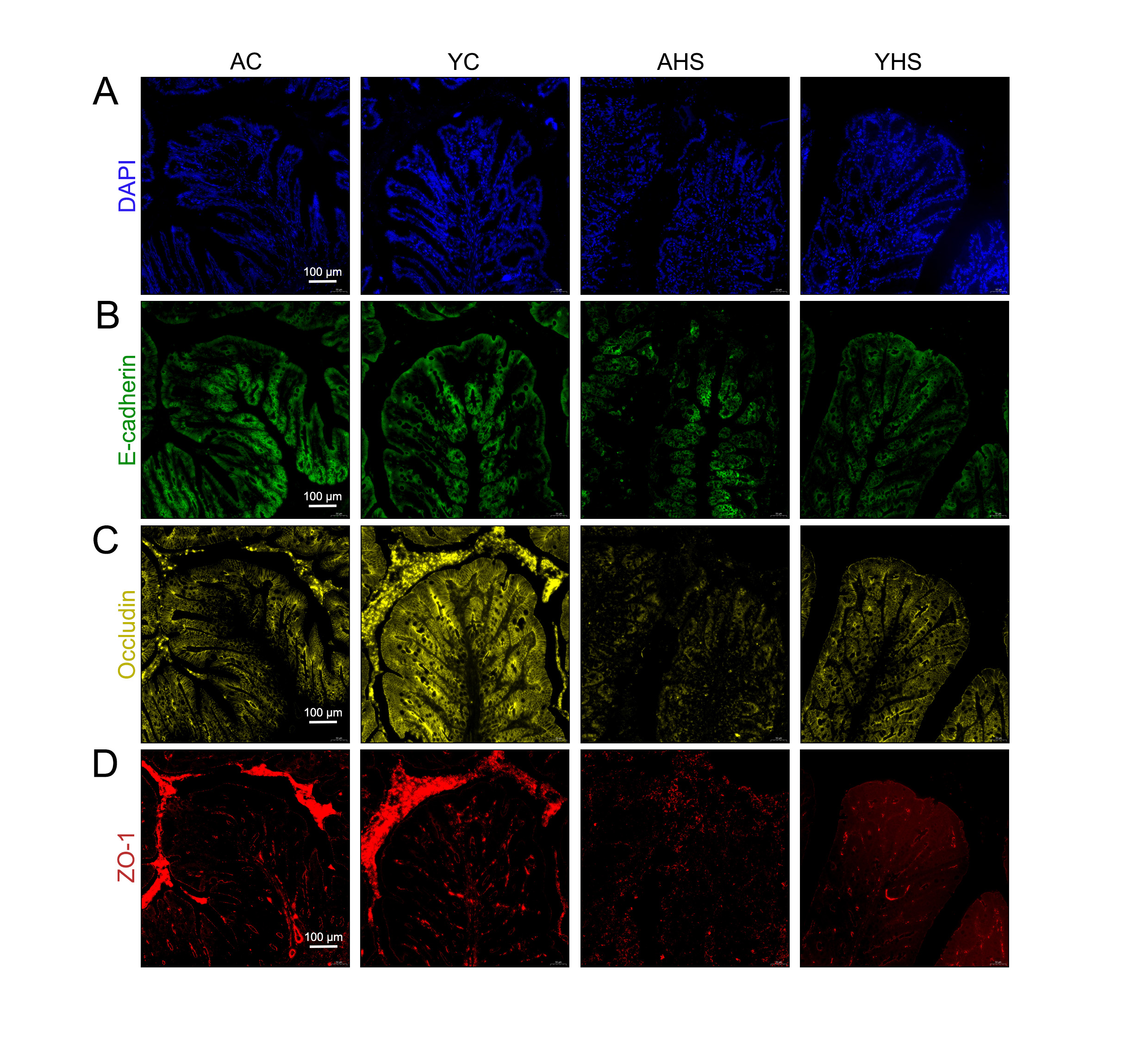

Supplement: Supplementary file 1 [file Image1.jpeg]
